# Child Allergic Symptoms and Well-Being at School: Findings from ALSPAC, a UK Cohort Study

**DOI:** 10.1371/journal.pone.0135271

**Published:** 2015-08-12

**Authors:** Alison Teyhan, Bruna Galobardes, John Henderson

**Affiliations:** School of Social and Community Medicine, University of Bristol, Bristol, United Kingdom; Swinburne University of Technology, AUSTRALIA

## Abstract

**Background:**

Eczema and asthma are common conditions in childhood that can influence children’s mental health. Despite this, little is known about how these conditions affect the well-being of children in school. This study examines whether symptoms of eczema or asthma are associated with poorer social and mental well-being in school as reported by children and their teachers at age 8 years.

**Methods:**

Participants were from the Avon Longitudinal Study of Parents and Children. Measures of child well-being in school were child-reported (n = 6626) and teacher reported (n = 4366): children reported on their enjoyment of school and relationships with peers via a self-complete questionnaire; teachers reported child mental well-being using the Strengths and Difficulties Questionnaire [binary outcomes were high ‘internalizing’ (anxious/depressive) and ‘externalizing’ (oppositional/hyperactive) problems (high was >90th percentile)]. Child rash and wheeze status were maternally reported and symptoms categorised as: ‘none’; ‘early onset transient’ (infancy/preschool only); ‘persistent’ (infancy/preschool and at school age); and ‘late onset’ (school age only).

**Results:**

Children with persistent (OR 1.29, 95% CI 1.02 to 1.63) and late onset (OR 1.48, 95% CI 1.02 to 2.14) rash were more likely to report being bullied, and children with persistent wheeze to feel left out (OR 1.42, 95% CI 1.10 to 1.84). Late onset rash was associated with high teacher-reported internalising behaviours (OR 1.61, 95% CI 1.02 to 2.54), and persistent rash with high externalising behaviours (OR 1.37, 95% CI 1.02 to 1.84). Child sleep and maternal mental health explained some of the associations with teacher-reported mental well-being.

**Conclusion:**

Symptoms of eczema or asthma can adversely affect a child’s social and mental well-being at primary school. This suggests interventions, such as additional support or education of peers, should begin at early stages in schooling.

## Introduction

Asthma and eczema are common childhood diseases, but little is known about how they affect children’s social and mental well-being in school. Studies which report increased levels of anxiety, depression, attention-deficit/hyperactivity disorder (ADHD), and behavioural problems in children with asthma [1–6] and eczema [7–10] have used mainly maternal reports of child mental well-being. Relatively little is known about the well-being of children with asthma or eczema in the school environment. Few studies have utilised teacher reports of child mental well-being; in a large UK study of children and adolescents, those with asthma had more teacher-reported emotional, peer, conduct and hyperactive problems than those without asthma [11]. However, in a Dutch case control study, children with asthma were rated by their teachers as being happier at school than children without asthma [12].

Both conditions have symptoms that can be noticeable to others, necessitate the use of medications, and result in school absence [13, 14]; thereby marking affected children as ‘different’ and potentially affecting their peer relationships. Bullying is common in childhood and has three key characteristics; repetition over time, harm, and unequal power. It can take many forms: physical (e.g. kicking); behavioural (e.g. being mean); verbal (e.g. derogatory remarks); or relational (e.g. disrupting social relationships between victim and peers) [15]. Children with chronic health conditions are known to be at increased risk of being a victim [15, 16], but there has been little research focused on asthma or eczema [17].

The few studies which have specifically considered whether asthma or eczema affect a child’s social well-being at school have had mixed results. In one Scandinavian study, parents reported that children with asthma, but not those with eczema, were more likely to be bullied [18]. However, in another Scandinavian study with child-reported measures, eczema was associated with being bullied in adolescent boys, but this association was not observed for girls, or for asthma for boys or girls [19]. In small studies from the UK and China, most children with eczema reported that their condition had a negative impact on their quality of life (due to itching, scratching and sleep disturbance), but only a minority reported that it made them feel embarrassed, affected their friendships, or caused them to be teased or bullied [20, 21]. In qualitative studies, bullying was frequently mentioned by 6–12 year old children with asthma [22], and patients with eczema reported being called names such as ‘leper’, peers thinking they were contagious, being made to feel like ‘a total freak’, and being socially excluded [23].

In this current study, we use data from a population-based birth cohort to examine the social and mental well-being at school of eight-year-old children with rash or wheeze. Our aims were to determine whether rash or wheeze were associated with (1) child reports of being bullied, left out, or unhappy at school and (2) teacher-reported child internalising (i.e. anxious and depressive) or externalising (i.e. oppositional and hyperactive) behaviours. We also considered whether child sleep or maternal mental health explained any of the association between child rash or wheeze and the teacher-reported mental well-being outcomes; it has previously been reported that adjustment for these factors attenuated associations between child rash and wheeze and maternally-reported child mental well-being [24].

## Methods

### Study population

The sample was from the population-based Avon Longitudinal Study of Parents and Children (ALSPAC) (www.bristol.ac.uk/alspac/) [25]. ALSPAC recruited pregnant women with expected dates of delivery between 1^st^ April 1991 and 31^st^ December 1992 and who lived in a defined geographical area (Avon, United Kingdom). There were 14,062 live births and 13,988 children alive at one year, of which 13,615 were singletons. We derived separate study samples for the analyses of the child-reported outcomes (n = 6,626) and teacher-reported outcomes (n = 4,366) due to a high proportion of children without teacher-reported outcome data.

### Ethics statement

Ethical approval for ALSPAC was obtained from the ALSPAC Ethics and Law Committee and the Local Research Ethics Committees (LREC). Full LREC details are available online (www.bristol.ac.uk/alspac/researchers/data-access/ethics/lrec-approvals/#d.en.16412). This study was approved by the ALSPAC Executive Committee. It involves secondary analysis of ALSPAC questionnaire data, which is fully anonymised before researchers can access or analyse it. No clinical records were used in this study.

### Measures

#### Child rash and wheeze

Child rash and wheeze symptoms were reported annually by the mother from when child was aged 6 months until age 7 years 7 months (S1 Table details question wording). The wheeze questions are consistent with those used by the International Study of Asthma and Allergies in Childhood and are a reliable way of diagnosing asthma in large epidemiological studies where direct physician assessment would not be feasible [26]. The rash questions relate to dry, itchy rashes in joints and creases, which likely represent eczema. Where questionnaires contained more than one question about wheeze or rash, a symptom was coded as being present if the mother reported yes to any one of the questions. The time periods were categorised as infancy (up to 18mths), preschool (18mths up to 4yrs 9mths) and school age (4yrs 9mths up to 7yrs 7mths). To be included in analyses, children had to have their rash and wheeze status reported in at least one questionnaire in each time period. For rash and wheeze separately, symptoms were categorised as being: ‘none’ (no symptoms in any time period); ‘early onset, transient’ (symptoms in infancy and/or preschool only); ‘persistent’ (symptoms in infancy and or preschool, and at school age); and ‘late onset’ (symptoms at school age only).

#### Teacher-reported child mental well-being

Teachers reported child mental well-being (at mean age 8.3 years) using the Goodman Strengths and Difficulties Questionnaire (SDQ) [27]. The peer and emotion subscales were summed to give an internalising problems score (range 0–20), and the hyperactivity and conduct subscales summed to give an externalising problems score (range 0–20) [28]. A higher score reflects more difficulties. For both outcomes, children were classified as having a high score (>90^th^ percentile) or not; the thresholds were ≥8 for internalising and ≥9 for externalising.

#### Child-reported school life variables

The children self-reported on their school life (mean age 8.2 years): happy at school (always, often, sometimes, never); left-out (never, sometimes, often, always); bullied (never, a little bit, quite a lot, all the time). As fewer than 2% of children reported the most negative category for each of these variables, binary variables were derived; two most positive categories versus two most negative categories for each outcome.

#### Other variables

Socioeconomic position (SEP) was measured with three separate variables: maternal education reported during pregnancy (degree, A level, O level, vocational or none), and financial difficulties (quartiles of score with range 0 to 40) and housing tenure (owned/mortgaged, rented privately, rented council, other) when child was 8 years. Child sleep disturbance was maternally reported when child aged 6yrs 9mths: wakes at night (no, once, twice or more). Maternal depression and anxiety when her child was aged 8 years; via the Edinburgh Postnatal Depression Scale [29] and the 20 trait anxiety items of the State-Trait Anxiety Inventory [30] respectively. Quartiles of depression and anxiety scores were derived as they were not normally distributed.

### Missing data

Each study sample comprised singleton children with complete outcome data, and exposure data (rash and wheeze status) reported by their mother at least once in each time period. Multiple imputation using chained equations (MICE) was used to replace missing exposure and confounder data with predictions based on information observed in the sample. Twenty-five imputed datasets were created for each sample. Missing data are summarised in S2 Table.

### Statistical analyses

Logistic regression was used for the child reported outcomes, and multilevel logistic regression (child clustered within school class) for the teacher-reported outcomes. First, the age and sex adjusted association between each of the covariates and the outcomes, and between the child-reported and teacher-reported outcomes, was examined. Next, the association between rash or wheeze status and each outcome was modelled, adjusted for age, sex, SEP and rash or wheeze status. Further models, which adjusted for child sleep and maternal anxiety and depression, were run where a significant association between rash or wheeze and a teacher-reported outcome was found, to determine if these factors explained the associations observed. Interaction terms were fitted to test whether the associations differed by sex; these were not significant.

## Results

### Sample characteristics

Rash and wheeze were common symptoms at school age, and were more likely to be persistent (i.e. symptoms started in infancy or preschool) than late onset (i.e. started for the first time at school-age) (Table 1). Almost 80% of the children reported being happy at school always or most of the time (Table 1). However, almost 10% reported frequently being left out or bullied.

**Table 1:** Study population characteristics.

		Child-reported outcomes sample	Teacher-reported outcomes sample
		Imputed, N = 6626	Imputed, N = 4366
Child rash	*None (%)*	28.9 (27.8–30.1)	29.0 (27.6–30.5)
	*Early onset transient (%)*	29.6 (28.5–30.8)	29.6 (28.1–31.1)
	*Persistent (%)*	33.8 (32.7–35.0)	33.6 (32.1–35.1)
	*Late onset (%)*	7.6 (6.9–8.3)	7.8 (6.9–8.7)
Child wheeze	*None (%)*	53.7 (52.4–54.9)	53.7 (52.2–55.3)
	*Early onset transient (%)*	27.7 (26.6–28.8)	27.3 (26.0–28.7)
	*Persistent (%)*	14.3 (13.5–15.2)	14.4 (13.4–15.5)
	*Late onset (%)*	4.3 (3.8–4.8)	4.5 (3.8–5.1)
Happy at school	*Sometimes or never (%)*	20.6 (19.6–21.5)	21.8 (20.3–23.3)
Left out	*Often or always (%)*	8.0 (7.3–8.6)	9.8 (8.6–10.9)
Bullied	*Quite a lot or all the time (%)*	8.2 (7.5–8.9)	9.3 (8.2–10.4)
		**N = 3273**	
Teacher-reported externalising score^1^	*High score (%)*	7.5 (6.6–8.4)	9.1 (8.3–10.0)
		**N = 3278**	
Teacher-reported internalising score^1^	*High score (%)*	6.7 (5.8–7.5)	7.6 (6.8–8.4)
Child sex	*Female (%)*	51.2 (50.0–52.4)	49.0 (47.6–50.5)
Child age at outcome	*Mean (years)*	8.2 (8.2–8.2)	8.3 (8.3–8.3)
Maternal age at delivery	*Mean (years)*	29.1 (29.0–29.2)	28.9 (28.7–29.0)
Maternal education	*Degree (%)*	16.4 (15.5–17.3)	14.3 (13.3–15.4)
	*A-level (%)*	26.2 (25.2–27.3)	26.0 (24.7–27.3)
	*O-Level (%)*	35.1 (33.9–36.2)	36.0 (34.5–37.4)
	*None/Vocational (%)*	22.3 (21.3–23.4)	23.7 (22.4–25.0)
Financial difficulties	*0 (%)*	52.9 (51.6–54.1)	52.5 (50.9–54.1)
	*1 (%)*	12.8 (12.0–13.7)	13.0 (11.9–14.1)
	*2–3 (%)*	17.5 (16.6–18.5)	17.8 (16.5–19.1)
	*4+ (%)*	16.8 (15.8–17.7)	16.7 (15.5–18.0)
Housing tenure	*Owned/Mortgaged (%)*	85.3 (84.4–86.2)	85.5 (84.4–86.6)
	*Rented—Private (%)*	3.5 (3.0–4.0)	3.3 (2.7–4.0)
	*Rented—Council/Housing Association (%)*	9.3 (8.6–10.0)	9.6 (8.6–10.5)
	*Other (%)*	1.9 (1.6–2.3)	1.6 (1.1–2.1)
Maternal anxiety (child aged 8 yrs)	*Lowest quartile (%)*	25.7 (24.6–26.8)	26.2 (24.7–27.6)
	*Highest quartile (%)*	24.1 (23.0–25.2)	23.8 (22.4–25.3)
Maternal depression (child aged 8yrs)	*Lowest quartile (%)*	30.2 (29.1–31.4)	30.6 (29.0–32.1)
	*Highest quartile (%)*	23.1 (22.1–24.2)	23.0 (21.5–24.5)
Child wakes at night	*Once (%)*	14.2 (13.3–15.1)	15.3 (14.2–16.5)
	*Twice or more (%)*	2.3 (1.9–2.7)	2.9 (2.3–3.4)

^1^ Teacher-reported outcomes were not imputed for the child-reported outcome study population

Correlates of both high internalising and externalising scores were being male, being younger, family financial difficulties, living in a rented council home, and maternal anxiety and depression (S3 Table). Similarly, boys, children from lower SEP families, and those whose mothers reported more depressive or anxious feelings, tended to self-report less favourably on their well-being at school (S3 Table). The child-reported and teacher-reported measures of well-being were associated; children who reported being bullied, left out, or unhappy at school were more likely to have a high teacher-reported internalising and externalising score (S4 Table).

### Association between rash, wheeze and child-reported outcomes

Children with persistent wheeze were more likely to report being left out at school than children who had never had wheeze [10.8% (95% CI 8.8%-12.8%) compared to 7.3% (6.5%-8.2%)], and more likely to report that they were never or only sometimes happy at school [23.4% (20.6% to 26.1%) compared to 19.4% (18.1% to 20.8%)]. Adjustment for measures of SEP attenuated these differences, but did not fully account for the greater likelihood of being left out (Table 2). Being bullied quite a lot or all the time was reported by 9.1% (7.9% to 10.3%) of children with persistent rash and 10.3% (7.4% to 13.2%) of children with late onset rash, compared to 7.4% (6.1% to 8.6%) of those with no rash at any age. These differences were not explained by SEP (Table 2).

**Table 2:** Association between child rash and wheeze and child-reported outcomes.

		OR (95% confidence interval)					
		Rash			Wheeze		
		Model 1^1^	Model 2^2^	Model 3^3^	Model 1^1^	Model 2^2^	Model 3^3^
Outcome	Rash or wheeze status	age, sex	age, sex, SEP	age, sex, SEP, wheeze	age, sex	age, sex, SEP	age, sex, SEP, rash
Happy at school sometimes or never	None (ref)						
	Early onset transient	1.04 (0.88–1.23)	1.05 (0.89–1.24)	1.04 (0.88–1.23)	1.08 (0.94–1.25)	1.04 (0.90–1.20)	1.04 (0.90–1.20)
	Persistent	1.05 (0.90–1.22)	1.06 (0.91–1.24)	1.04 (0.88–1.22)	1.23 (1.04–1.47)*	1.15 (0.96–1.37)	1.14 (0.95–1.36)
	Late onset	0.98 (0.76–1.28)	0.99 (0.76–1.29)	0.98 (0.76–1.28)	1.22 (0.90–1.66)	1.17 (0.86–1.60)	1.17 (0.86–1.59)
Left out often or always	None (ref)						
	Early onset transient	0.92 (0.72–1.19)	0.92 (0.71–1.18)	0.89 (0.69–1.15)	1.07 (0.86–1.34)	1.04 (0.83–1.29)	1.04 (0.83–1.30)
	Persistent	1.07 (0.85–1.34)	1.07 (0.85–1.34)	1.01 (0.80–1.28)	1.51 (1.18–1.94)*	1.42 (1.11–1.82)*	1.42 (1.10–1.84)*
	Late onset	1.18 (0.81–1.71)	1.18 (0.81–1.71)	1.17 (0.80–1.70)	0.91 (0.54–1.53)	0.85 (0.51–1.44)	0.85 (0.50–1.43)
Bullied often or always	None (ref)						
	Early onset transient	1.02 (0.79–1.32)	1.02 (0.78–1.32)	1.01 (0.78–1.32)	1.08 (0.88–1.34)	1.03 (0.83–1.28)	1.02 (0.82–1.26)
	Persistent	1.29 (1.03–1.63)*	1.31 (1.04–1.66)*	1.29 (1.02–1.63)*	1.30 (1.01–1.68)*	1.17 (0.90–1.51)	1.11 (0.85–1.45)
	Late onset	1.46 (1.01–2.11)*	1.49 (1.03–2.15)*	1.48 (1.02–2.14)*	1.19 (0.75–1.89)	1.12 (0.71–1.79)	1.07 (0.67–1.71)

^1^Adjusted for child age and sex

^2^ Adjusted for variables in model 1 plus: maternal age, maternal education, financial difficulties, housing tenure

^3^ Adjusted for variables in model 2 plus: wheeze (in rash model) or rash (in wheeze model)

*compared to reference category, p<0.05

### Association between rash, wheeze and teacher-reported outcomes

Child wheeze symptoms were not associated with teacher-reported internalising or externalising behaviours (Table 3). For rash, children with late onset symptoms were the most likely to have a high teacher-reported externalising (10.7%, 6.9% to 14.5%) and internalising (10.0%, 6.6% to 13.4%) score, and those who had never had symptoms were the least likely to have a high score [externalising (8.5%, 95% CI 6.9% to 10.1%); internalising (6.7%, 5.3% to 8.1%)]. After adjustment for SEP and wheeze status, late onset rash remained associated with a high internalising score (Table 3). For the externalising outcome, the odds of having a high score increased for the persistent rash group relative to the never rash group after adjustment for SEP and wheeze status (Table 3). Odds ratios were highest for the late onset group but confidence intervals were wide.

**Table 3:** Association between child rash and wheeze and teacher-reported outcomes.

		OR (95% confidence interval)					
		Rash			Wheeze		
		Model 1^1^	Model 2^2^	Model 3^3^	Model 1^1^	Model 2^2^	Model 3^3^
Outcome	Rash or wheeze status	age, sex	age, sex, SEP	age, sex, SEP, wheeze	age, sex	age, sex, SEP	age, sex, SEP, rash
High internalising	None (ref)						
	Early onset transient	1.29 (0.95–1.76)	1.30 (0.96–1.78)	1.31 (0.96–1.78)	1.11 (0.84–1.47)	1.05 (0.79–1.39)	1.04 (0.78–1.37)
	Persistent	1.11 (0.81–1.53)	1.11 (0.80–1.53)	1.13 (0.81–1.58)	0.96 (0.67–1.37)	0.89 (0.62–1.28)	0.89 (0.62–1.28)
	Late onset	1.64 (1.05–2.59)*	1.61 (1.02–2.53)*	1.61 (1.02–2.54)*	0.83 (0.43–1.63)	0.80 (0.41–1.56)	0.80 (0.40–1.57)
High externalising	None (ref)						
	Early onset transient	1.22 (0.91–1.65)	1.29 (0.95–1.74)	1.30 (0.95–1.76)	1.19 (0.92–1.55)	1.07 (0.82–1.39)	1.05 (0.81–1.37)
	Persistent	1.20 (0.90–1.60)	1.33 (0.99–1.77)	1.37 (1.02–1.84)*	0.93 (0.67–1.30)	0.85 (0.60–1.19)	0.81 (0.57–1.14)
	Late onset	1.52 (0.92–2.49)	1.56 (0.93–2.59)	1.56 (0.93–2.60)	0.96 (0.53–1.74)	0.92 (0.50–1.69)	0.90 (0.49–1.67)

^1^Adjusted for child age and sex

^2^ Adjusted for variables in model 1 plus: maternal age, maternal education, financial difficulties, housing tenure

^3^ Adjusted for variables in model 2 plus: wheeze (in rash model) or rash (in wheeze model)

*compared to reference category, p<0.05

The association between late onset rash and high internalising score was slightly attenuated by further adjustment for child sleep (OR1.56, 95% CI 0.99 to 2.48), or maternal anxiety and depression (1.56, 0.98 to 2.47), or both child sleep and maternal anxiety and depression (1.52, 0.96–2.42). The association between persistent rash and high externalising score was unaffected by adjustment for child sleep (1.36, 1.01–1.83) but was attenuated by adjustment for maternal anxiety and depression (1.32, 0.98–1.78).

## Discussion

Children with rash were more likely to report being bullied, and those with wheeze to report being left out, at the age of 8 years in our population-based cohort. However, the vast majority of children with rash or wheeze reported positively on their school life. Children with rash, but not with wheeze, had more internalising and externalising teacher-reported behaviours.

Persistent wheeze was associated with feeling left out at school in our study. Being left out can be a type of relational bullying [15]. However, the children may have felt left out due to their condition preventing them from participating in activities, or causing school absence, rather than as a result of bullying. Either way, our results suggest children with persistent wheeze have more difficulties forming friendships at school. This is consistent with previous studies that have identified parent-reported [31, 32] and child-reported [22, 33, 34] friendship difficulties in children with asthma; not being able to play with friends, not having a group of friends to hang around with, feeling isolated and lonely, and being left out of social activities. There is a paucity of research on bullying in children with asthma, and results have been mixed. Bullying was frequently mentioned by 6–12 year old children with asthma in a qualitative study [22], however in a large cohort study there was no association between asthma and child-reported bullying at the age of 15 years [19]. Two cohort studies did report an increased risk of being bullied in children with asthma, but they used parent-reported measures of bullying and included a wide age range from infancy to 17 years [1, 18].

Children with rash at school age in our study reported an increased risk of being bullied. Although bullying is frequently appearance related, there has been little previous research on bullying in children or adolescents affected by skin disease [17]. In a large Norwegian school-based study of 15 year olds, boys who reported being bullied were more likely to have eczema than boys who were not bullied, but the prevalence of bullying was not reported and no association was observed for girls [19]. Three small studies which used the Children’s Dermatology Life Quality Index did report the prevalence of teasing, bullying and friendship problems due to eczema but had no controls with which to compare the prevalence rates: less than 10% of 43 children aged 5–10 years in Hong Kong said their eczema caused them to be bullied, or affected friendships, ‘very much or a lot’ [21]. Of 78 children aged 6–11 years in London and Wales, 17% reported being teased or bullied and 15% that it affected friendships [20]. Proportions were higher in a US study of 178 children aged 5–15 years, but this is likely due to ‘a little affected’ being combined with ‘very much or a lot’ in this study; 30% reported that their eczema affected friendships and 50% that it caused them to be teased or bullied [35].

Taken together, results from the previous studies are generally in concordance with ours; having eczema or asthma symptoms can increase the likelihood of a child being bullied or left out at school, but most of the children affected by these conditions are not bullied and do not have friendship problems at school. While this is a positive finding, the children in our sample were only eight years old, and it is known that most types of bullying increase with age [15].

We were able to investigate teacher-reported measures of child mental well-being. Child rash, particularly persistent rash, was associated with an increased likelihood of having a high externalising score. Late onset rash was associated with a high internalising score. In a previous study using ALSPAC data, children with rash were more likely to have a high maternal-reported internalising and externalising score [24]; this concordance suggests the behaviour of children with rash is affected in both their home and school life. We found no association between child wheeze and teacher-reported mental well-being, whereas the previous ALSPAC study found persistent wheeze to be associated with both maternal-reported internalising and externalising outcomes [24]. This could mean wheeze has a greater impact on a child’s behaviour at home than at school. However, in a large UK study, children aged 5–15 years with asthma, particularly those in poor health, had more teacher-reported internalising and externalising problems, than their healthy peers [11]. Our discordant findings could be due to maternal reporting bias, or to teachers not noticing or interpreting a child’s behaviour in the same way as the mothers.

Adjustment for maternal anxiety and depression attenuated associations between persistent rash and externalising problems, and late onset rash and internalising problems. This concurs with the previous ALSPAC study which found maternal anxiety and depression accounted for the association between child rash and maternal-reported internalising symptoms and partly for externalising symptoms [24] and previous studies which found children with eczema and asthma are more likely to have a mother with anxiety or depression compared with healthy children [36–39].

Despite sleep quality being considered a key factor in the association between eczema, asthma and behavioural problems in children [8, 9, 40, 41], it only explained some of the association between late onset rash and teacher-reported internalising problems, and did not alter the association between persistent rash and externalising problems. However, child sleep quality was measured at age 6 years 9 months and may not reflect sleep quality at the outcome time point, particularly if rash or wheeze symptoms changed after sleep quality was measured. Although rash and wheeze are likely to represent eczema and asthma in school-aged children, these symptoms are less specific in younger children and so not all reported rash and wheeze will have been due to eczema or asthma. We also had no measure of disease severity. Our child and teacher reported outcomes measured two different aspects of child well-being at school. As both were measured at the same point in time (child aged 8), it was not possible to examine how one influenced the other (i.e. did bullying result in a change in behaviour, or vice versa). Additionally, the children in our sample were at primary school in the late 1990s and early 2000s; school policies on bullying and child well-being may have changed over time. Further research on how the relationship between rash and wheeze and school well-being changes with age could help determine if there is a high-risk period, or identify the most appropriate age for interventions to prevent the initiation of chronic-disease related bullying.

Few previous studies on the well-being of children with asthma or eczema have included child or teacher reports. We have shown that having rash or wheeze can adversely affect a child’s social and mental well-being at primary school. The children in our sample were relatively young compared to many previous studies; our results thus signal the importance of recognising that these chronic conditions have social and behavioural comorbidities even at this young age. Furthermore, they suggest that any intervention, such as additional support at school or education of peers, should begin early.

## Supporting Information

S1 Table Summary of ALSPAC questionnaire items on rash and wheeze. (DOCX)

S2 Table Description of missing data. (DOCX)

S3 Table Association between covariates and teacher and child reported outcomes. (DOCX)

S4 Table Association between child-reported school life and teacher-reported internalising and externalising behaviours. (DOCX)
