# Supplementary material for: Child Allergic Symptoms and Well-Being at School: Findings from ALSPAC, a UK Cohort Study
Source: PLoS One. 2015 Aug 12;10(8):e0135271. doi: 10.1371/journal.pone.0135271 (PMC4534318; doi:10.1371/journal.pone.0135271)
Supplement: S1 Table — (DOCX) [file pone.0135271.s001.docx]

**S1 Table: Summary of ALSPAC questionnaire items on rash and wheeze**

| **Child’s age when questionnaire sent to mother** | **“Has your child had....”**  (since birth for 6mth questionnaire, since the previous questionnaire time-point for all others) |
| --- | --- |
| **Rash** |  |
| 6mths, 18mths | 1. A rash in the joints and creases of his/her body (e.g. behind the knees, under the arms)?  2. An itchy, dry, oozing or crusted rash on the face, forearms or shins? |
|  |  |
| 2yrs 6mths, 3yrs 6mths, 4yrs 9mths, 5yrs 9mths, 6yrs 9mths | 1. An itchy, dry skin rash in the joints and creases of his/her body (e.g. behind the knees, elbows, under the arms)?  2. An itchy, dry rash on his hands?  3. An itchy, dry rash on his feet? |
|  |  |
| 7yrs 7mths | 1. A rash? |
| **Wheeze** |  |
| 6mths, 18mths, 2yrs 6mths, 3yrs 6mths, 4yrs 9mths, 5yrs 9mths, 6yrs 9mths | 1. Wheezing with whistling on his chest when he/she breathed?  2. Wheezing? |
|  |  |
| 7yrs 7mths | 1. Wheezing? |
