# Supplementary material for: Child Allergic Symptoms and Well-Being at School: Findings from ALSPAC, a UK Cohort Study
Source: PLoS One. 2015 Aug 12;10(8):e0135271. doi: 10.1371/journal.pone.0135271 (PMC4534318; doi:10.1371/journal.pone.0135271)
Supplement: S2 Table — (DOCX) [file pone.0135271.s002.docx]

**S2 Table: Description of missing data**

|  | |  | **Child-reported outcome sample** | **Teacher-reported outcome sample** |
| --- | --- | --- | --- | --- |
|  | |  | n (%) missing | n (%) missing |
|  | |  | (6626=100%)^1^ | (4366=100%)^2^ |
| Teacher-reported internalising score | |  | 3348 (50.5) | 0 |
| Teacher-reported externalising score | |  | 3353 (50.6) | 0 |
|  | |  |  |  |
| Happy at school | |  | 0 | 1048 (24.0) |
| Left out | |  | 0 | 1056 (24.2) |
| Bullied | |  | 0 | 1043 (23.9) |
| Own behaviour | |  | 0 | 1058 (24.2) |
|  | | |  |  |
| Wheeze | | |  |  |
|  | 6mths | | 201 (3.0) | 160 (3.7) |
|  | 18mths | | 147 (2.2) | 136 (3.1) |
|  | 2yrs 6mths | | 323 (4.9) | 286 (6.6) |
|  | 3yrs 6mths | | 310 (4.7) | 253 (5.8) |
|  | 4yrs 9mths | | 325 (4.9) | 314 (7.2) |
|  | 5yrs 9mths | | 426 (6.4) | 417 (9.6) |
|  | 6yrs 9mths | | 496 (7.5) | 518 (11.9) |
|  | 7yrs 7mths | | 442 (6.7) | 631 (14.5) |
|  |  | |  |  |
| Rash | | |  |  |
|  | 6mths | | 239 (3.61) | 182 (4.2) |
|  | 18mths | | 154 (2.3) | 136 (3.1) |
|  | 2yrs 6mths | | 373 (5.6) | 311 (7.1) |
|  | 3yrs 6mths | | 305 (4.6) | 241 (5.5) |
|  | 4yrs 9mths | | 689 (10.4) | 530 (12.1) |
|  | 5yrs 9mths | | 815 (12.3) | 653 (15.0) |
|  | 6yrs 9mths | | 712 (10.8) | 656 (15.0) |
|  | 7yrs 7mths | | 452 (6.8) | 633 (14.5) |
|  |  | |  |  |
| Child sex | | | 0 | 0 |
| Mother’s age at delivery | | | 0 | 0 |
| Housing tenure | | | 458 (6.9) | 597 (13.7) |
| Maternal education | | | 136 (2.1) | 119 (2.7) |
| Financial difficulties | | | 576 (8.7) | 672 (15.4) |
| Maternal anxiety during pregnancy | | | 616 (9.3) | 431 (9.87) |
| Maternal depression during pregnancy | | | 526 (7.9) | 377 (8.63) |
| Maternal anxiety when child aged 8 years | | | 745 (11.2) | 1145 (26.2) |
| Maternal depression when child aged 8 years | | | 418 (6.3) | 953 (21.8) |
| Child sleep | | | 555 (8.4) | 566 (13.0) |

^1^ The child-reported study sample was defined as singleton children with complete outcome data who had rash and wheeze status reported at least once in each time period (infancy, preschool, school age), n=6626.

^2^ The teacher-reported study sample was defined as singleton children with complete outcome data who had rash and wheeze status reported at least once in each time period (infancy, preschool, school age), n=4366.
