# Supplementary material for: Child Allergic Symptoms and Well-Being at School: Findings from ALSPAC, a UK Cohort Study
Source: PLoS One. 2015 Aug 12;10(8):e0135271. doi: 10.1371/journal.pone.0135271 (PMC4534318; doi:10.1371/journal.pone.0135271)
Supplement: S3 Table — (DOCX) [file pone.0135271.s003.docx]

**S3 Table: Association between covariates and teacher and child reported outcomes**

|  |  | | OR (95% CI)^1^ | | OR (95% CI)^1^ | | |  |
| --- | --- | --- | --- | --- | --- | --- | --- | --- |
|  |  | | High internalising | High externalising | Happy school sometimes/never | Left out | Bullied |  |
| **Sex (Ref: Male)** | | |  |  |  |  |  |  |
|  | Female | | 0.69 (0.54-0.86)* | 0.19 (0.14-0.24)* | 0.81 (0.72-0.92)* | 0.96 (0.80-1.15) | 0.66 (0.55-0.79)* |  |
| **Age (yrs)** | | | 0.69 (0.47-1.01) | 0.49 (0.34-0.71)* | 1.03 (0.99-1.06) | 1.05 (1.00-1.10)* | 1.02 (0.98-1.07) |  |
| **Maternal education (Ref: Degree)** | | |  |  |  |  |  |  |
|  | A-level | | 0.91 (0.62-1.34) | 2.32 (1.43-3.75)* | 1.14 (0.94-1.40) | 1.13 (0.84-1.51) | 0.95 (0.71-1.28) |  |
|  | O-level | | 0.93 (0.64-1.35) | 2.28 (1.43-3.64)* | 1.26 (1.04-1.52)* | 1.14 (0.87-1.50) | 1.14 (0.87-1.50) |  |
|  | None/vocational | | 1.06 (0.72-1.57) | 4.33 (2.71-6.93)* | 1.57 (1.28-1.91)* | 1.11 (0.82-1.50) | 1.46 (1.10-1.95)* |  |
| **Financial difficulties (Ref: None)** | | |  |  |  |  |  |  |
|  | 1 | | 1.40 (0.97-2.02) | 0.85 (0.55-1.30) | 1.27 (1.05-1.55)* | 0.97 (0.71-1.32) | 1.07 (0.79-1.46) |  |
|  | 2-3 | | 1.26 (0.89-1.79) | 1.44 (1.04-1.99)* | 1.66 (1.40-1.96)* | 1.35 (1.05-1.72)* | 1.28 (1.00-1.66) |  |
|  | 4+ | | 1.52 (1.09-2.14)* | 1.87 (1.37-2.54)* | 1.83 (1.55-2.17)* | 1.69 (1.33-2.13)* | 1.85 (1.47-2.33)* |  |
| **Housing tenure (Ref: owned/mortgaged)** | | |  |  |  |  |  |  |
|  | | Privately rented | 0.71 (0.31-1.64) | 1.27 (0.68-2.40)* | 1.07 (0.77-1.50) | 2.02 (1.34-3.06)* | 1.28 (0.79-2.07) |  |
|  | | Council/Housing authority rented | 2.01 (1.41-2.86)* | 3.10 (2.24-4.29)* | 1.52 (1.25-1.86)* | 1.56 (1.17-2.08)* | 2.37 (1.85-3.03)* |  |
|  | | Other | 1.52 (0.52-4.43) | 0.45 (0.06-3.25) | 1.19 (0.76-1.86) | 1.56 (0.82-2.96) | 1.63 (0.90-2.97) |  |
| **Maternal anxiety when child 8yrs (Ref: Q1, low)** | | |  |  |  |  |  |  |
|  | Q2 | | 0.99 (0.64-1.53) | 0.90 (0.62-1.30) | 1.14 (0.95-1.37) | 1.29 (0.96-1.74) | 0.96 (0.72-1.28) |  |
|  | Q3 | | 1.30 (0.87-1.94) | 1.73 (1.20-2.50)* | 1.30 (1.08-1.57)* | 1.41 (1.05-1.90)* | 1.42 (1.08-1.87)* |  |
|  | Q4, high | | 1.82 (1.26-2.64)* | 1.53 (1.04-2.25)* | 1.70 (1.42-2.04)* | 2.19 (1.67-2.87)* | 1.87 (1.44-2.42)* |  |
| **Maternal depression when child 8 yrs (Ref: Q1, low)** | | |  |  |  |  |  |  |
|  | Q2 | | 1.41 (0.98-2.02)* | 1.33 (0.94-1.89) | 0.91 (0.75-1.09) | 1.07 (0.82-1.40) | 1.13 (0.86-1.47) |  |
|  | Q3 | | 1.44 (0.98-2.14)* | 1.98 (1.41-2.79)* | 1.34 (1.13-1.60)* | 1.18 (0.90-1.54) | 1.43 (1.10-1.87)* |  |
|  | Q4, high | | 1.79 (1.25-2.58)* | 1.67 (1.16-2.40)* | 1.44 (1.22-1.70)* | 1.74 (1.37-2.23)* | 1.76 (1.38-2.25)* |  |
| **Child wakes at night (Ref: No)** | | |  |  |  |  |  |  |
|  | Once | | 1.34 (0.95-1.89) | 1.21 (0.88-1.66) | 1.05 (0.88-1.25) | 1.09 (0.84-1.43) | 1.22 (0.94-1.58) |  |
|  | Twice of more | | 2.95 (1.66-5.22)* | 1.85 (0.98-3.51) | 1.19 (0.79-1.79) | 0.98 (0.50-1.90) | 2.33 (1.43-3.80)* |  |

^1^All models adjusted for child sex and age

*compared to reference category, p<0.05
