# Supplementary material for: Child Allergic Symptoms and Well-Being at School: Findings from ALSPAC, a UK Cohort Study
Source: PLoS One. 2015 Aug 12;10(8):e0135271. doi: 10.1371/journal.pone.0135271 (PMC4534318; doi:10.1371/journal.pone.0135271)
Supplement: S4 Table — (DOCX) [file pone.0135271.s004.docx]

**S4 Table: Association between child-reported school life and teacher-reported internalising and externalising behaviours**

|  |  | OR (95% CI)^1^ | |  |
| --- | --- | --- | --- | --- |
|  |  | High internalising | High externalising |  |
| **Happy at school (Ref: Always)** | |  |  |  |
|  | Often | 1.09 (0.80-1.50) | 0.96 (0.69-1.33) |  |
|  | Sometimes/Never | 1.77 (1.26-2.48)* | 2.33 (1.68-3.23)* |  |
| **Left Out (Ref: Never)** | |  |  |  |
|  | Sometimes | 1.51 (1.06-2.14)* | 0.93 (0.69-1.27) |  |
|  | Often/Always | 3.62 (2.30-5.71)* | 2.23 (1.43-3.47)* |  |
| **Bullied (Ref: Never)** | |  |  |  |
|  | Little bit | 1.45 (1.06-2.00)* | 1.84 (1.37-2.49)* |  |
|  | Quite a lot/all the time | 3.22 (2.16-4.79)* | 4.93 (3.22-7.56)* |  |
| **Behaviour school (Ref: Well, always)** | |  |  |  |
|  | Well, most of the time | 1.22 (0.92-1.60) | 5.47 (3.94-7.59)* |  |
|  | Badly most or all of the time | 1.35 (0.47-3.86) | 14.63 (7.05-30.37)* |  |

^1^All models adjusted for child sex and age, n=4366

*compared to reference category, p<0.05
